# Liposomes Targeting P21 Activated Kinase-1 (PAK-1) and Selective for Secretory Phospholipase A_2_ (sPLA_2_) Decrease Cell Viability and Induce Apoptosis in Metastatic Triple-Negative Breast Cancer Cells

**DOI:** 10.3390/ijms21249396

**Published:** 2020-12-10

**Authors:** Wided Najahi-Missaoui, Nhat D. Quach, Payaningal R. Somanath, Brian S. Cummings

**Affiliations:** 1 Department of Pharmaceutical and Biomedical Sciences, College of Pharmacy, University of Georgia, Athens, GA 30602, USA; mwided@uga.edu; 2 Department of Molecular Pharmacology, Physiology & Biotechnology, Brown University, Providence, RI 02906, USA; nhat_quach@brown.edu; 3 Clinical and Experimental Therapeutics, University of Georgia and Charlie Norwood VA Medical Center, Augusta, GA 30912, USA; sshenoy@augusta.edu; 4 Department of Medicine and Cancer Center, Augusta University, Augusta, GA 30912, USA; 5 Interdisciplinary Toxicology Program, University of Georgia, Athens, GA 30602, USA

**Keywords:** triple-negative breast cancer, SSL-IPA-3, SPRL-IPA-3, cell toxicity, cell death

## Abstract

P21 activated kinases (or group I PAKs) are serine/threonine kinases whose expression is altered in prostate and breast cancers. PAK-1 activity is inhibited by the small molecule “Inhibitor targeting PAK-1 activation-3” (IPA-3), which has selectivity for PAK-1 but is metabolically unstable. Secretory Group IIA phospholipase A_2_ (sPLA_2_) expression correlates to increased metastasis and decreased survival in many cancers. We previously designed novel liposomal formulations targeting both PAK-1 and sPLA_2,_ called Secretory Phospholipase Responsive liposomes or SPRL-IPA-3, and demonstrated their ability to alter prostate cancer growth. The efficacy of SPRL against other types of cancers is not well understood. We addressed this limitation by determining the ability of SPRL to induce cell death in a diverse panel of cells representing different stages of breast cancer, including the invasive but non-metastatic MCF-7 cells, and metastatic triple-negative breast cancer (TNBC) cells such as MDA-MB-231, MDA-MB-468, and MDA-MB-435. We investigated the role of sPLA_2_ in the disposition of these liposomes by comparing the efficacy of SPRL-IPA-3 to IPA-3 encapsulated in sterically stabilized liposomes (SSL-IPA-3), a formulation shown to be less sensitive to sPLA_2_. Both SSL-IPA-3 and SPRL-IPA-3 induced time- and dose-dependent decreases in MTT staining in all cell lines tested, but SPRL-IPA-3-induced effects in metastatic TNBC cell lines were superior over SSL-IPA-3. The reduction in MTT staining induced by SPRL-IPA-3 correlated to the expression of Group IIA sPLA_2_. sPLA_2_ expression also correlated to increased induction of apoptosis in TNBC cell lines by SPRL-IPA-3. These data suggest that SPRL-IPA-3 is selective for metastatic TNBC cells and that the efficacy of SPRL-IPA-3 is mediated, in part, by the expression of Group IIA sPLA_2_.

## 1. Introduction

Breast cancer is one of the most common invasive cancers affecting women worldwide and is a leading cause of cancer-related deaths [1]. The median age at the time of breast cancer diagnosis is 61 years, and about 20% of breast cancer patients are diagnosed before the age of 50. In general, 60% of breast cancers are diagnosed at a localized stage that is treated with breast-conserving surgery (BCS) or mastectomy [2]. The 5-year relative survival rate for women diagnosed with localized breast cancer is 98.6%, but survival declines to 83.8% for regional stages and 23.3% for distant stages [3]. Approximately 10% to 20% of breast cancer patients have triple-negative breast cancer (TNBC) and are usually diagnosed at late stages of the disease [2,4]. The survival rate among these patients is considerably lower despite the aggressive combination of surgery, radiation, and chemotherapy, demonstrating an urgent need for novel therapeutic approaches to advance this field [5,6].

Breast cancer is heterogeneous and is characterized by altered expression and/or activity of different protein kinases such as the p21-activated kinase-1 (PAK-1) [7]. PAK-1 is a serine/threonine kinase that belongs to the PAK family, which is classified into two groups: group I with PAK-1, 2, 3, and group II with PAK-4, 5, and 6. Group I PAKs are effectors of Cdc42 (cell division control protein 42) and Rac 1 (Ras-related C3 botulinum toxin substrate 1) [7,8,9]. PAK-1 (and other PAKs) are overexpressed in various cancers including prostate and breast cancers [10,11,12,13,14]. PAKs are involved in multiple cell signaling pathways that promote cell survival and proliferation, which makes them potential therapeutic targets in cancer [8,15]. PAK-1 has been shown to play a major role in regulating the cytoskeletal organization and cell migration [16]. More than 50% of human breast cancers display altered expression and activity of PAK-1, which correlated with increased survival of cancer cells, suggesting PAK-1 as a potential therapeutic target [17].

Several studies have tested the therapeutic effect of PAK-1 inhibitors on cancer cell growth [18,19]. Unfortunately, such studies are hampered by the limited effective and selective PAK-1 inhibitors [18,20]. One inhibitor, IPA-3 (Inhibitor targeting P21 Activated Kinase-1 activation-3), was identified as a small molecule allosteric inhibitor of PAK-1 after general screening for inhibitors of Cdc42 activation of PAKs [21]. IPA-3 targets the auto-regulatory mechanism of PAK-1. It binds to the PAK-1 regulatory domain and prevents its GTPase binding and subsequent activation. This unique mechanism of action accounts for the high selectivity of IPA-3 to PAK-1 [20,22,23]. 

A major limitation of IPA-3 is its stability, which is believed to result in a short half-life in vivo due to the reduction of its sulfhydryl moiety [9,24]. It also has some potential off-target effects [9,19,21,25]. We addressed this limitation by developing a novel liposome formulation encapsulating IPA-3 into stealth or sterically stabilized liposomes (SSL), which can improve the solubility of many drugs and can provide slow and sustained release of encapsulated drugs [26,27,28]. In addition, SSLs are known to alter the pharmacokinetic profile of encapsulated drugs and improve their pharmacological activities [29,30,31]. Our studies showed that SSL-IPA-3 improved the stability of IPA-3 and improved its efficacy with regard to inhibiting prostate cancer growth both in vitro and in vivo [32,33,34]. While these studies focused on prostate cancer, we have also previously shown that the ability of SSL-IPA-3 to decrease cell growth was dependent on the expression of PAK-1 in a diverse set of breast cancer cells. 

In an effort to further increase the efficacy of SSL, glycerophospholipids selectively cleaved by Group IIA phospholipase A_2_ (sPLA_2_) were incorporated into the formulation. This resulted in a novel formulation containing an increased level of 1,2-distearoyl-*sn*-glycero-3-phosphoethanolamine (DSPE). This formulation called secretory phospholipase responsive liposomes, or SPRL, was demonstrated to have increased efficacy in vitro and in vivo [34,35]. We further demonstrated that the increased efficacy was due, in part, to the increased selectivity for sPLA_2_ [34], which was conferred by the inclusion of the anionic DSPE (Table 1) [36]. The effect of these molecular differences between SSL and SPRL was tested in vitro and in vivo where SPRL had a greater effect on limiting the growth of prostate cancer xenografts compared to the conventional SSL [34,35].

The above studies suggest the clinical utility of SSL- and SPLR-IPA-3 for prostate cancer. Our studies also suggest that SSL-IPA-3 can inhibit breast cancer cell growth. However, SPRL has never been validated with a different drug in non-prostate cancer cell lines. Studies are also limited with regards to the molecular determinants of SPRL efficacy. This prospect is exciting as we have previously shown that SPRL containing doxorubicin, a standard treatment for breast cancer, demonstrated improved efficacy over SSL in xenograft models of prostate cancer [35]. Besides, a recent study assessing the expression of Group IIA PLA_2_ in a cohort of advanced breast cancer patients reported a large proportion to have high levels of expression in both primary tumors and metastasis [37]. Thus, this study tested the hypothesis that SPRL-IPA-3 displays an enhanced ability to inhibit breast cancer cell growth and induce cell death as compared to free IPA-3 or IPA-3 encapsulated in SSL.

## 2. Results

### 2.1. Characterization of SSL-IPA-3 and SPRL-IPA-3

**Figure 1:**
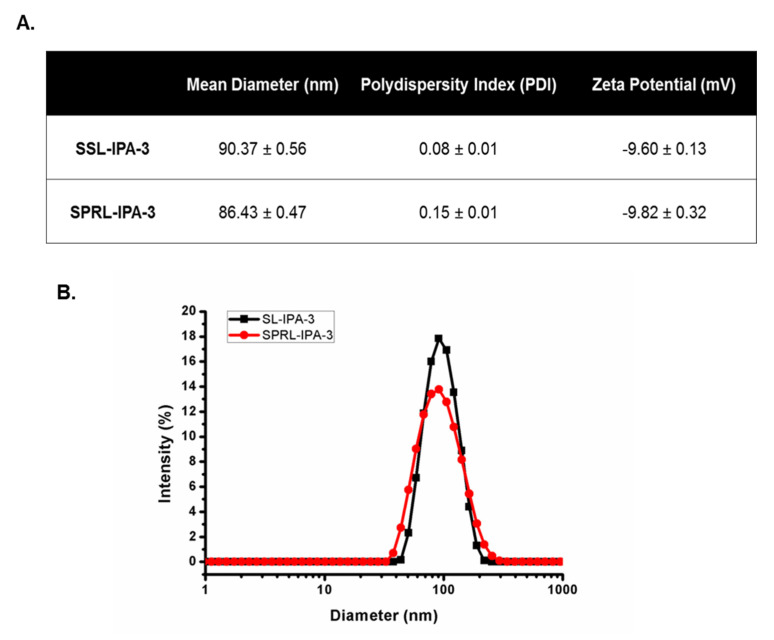
Characterization of SSL-IPA-3 and SPRL-IPA-3. (**A**) Table summarizing the hydrodynamic diameter, polydispersity index (PDI), and zeta potential of SSL-IPA-3 and SPRL-IPA-3 as determined using dynamic light scattering (DLS). (**B**) The size distribution of the liposomal suspensions (SSL-IPA-3 in black and SPRL-IPA-3 in red) as determined using dynamic light scattering (DLS). Data are representative of at least three different measurements.

SSL-IPA-3 and SPRL-IPA-3 had average hydrodynamic diameters of approximately 90 nm and polydispersity index values less than 0.2, indicating uniform size distribution (Figure 1). SSL-IPA-3 and SPRL-IPA-3 were negatively charged, with zeta potentials of −9.60 and −9.82 mV, respectively. Tandem electron microscopy (TEM) images showed that both SSL-IPA-3 and SPRL-IPA-3 liposomes were spherical with similar sizes (Supplementary Figure S1).

### 2.2. Effect of IPA-3 Encapsulated Liposomes (SSL-IPA-3) on Breast Cancer Cell Viability

**Figure 2:**
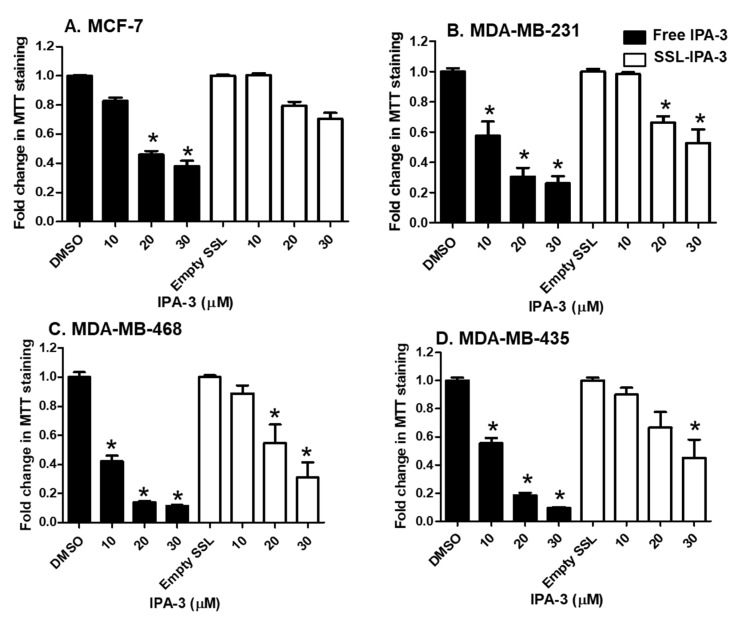
Effect of free IPA-3 and SSL-IPA-3 on MTT staining in human breast cancer MCF-7 (**A**), MDA-MB-231 (**B**), MDA-MB-468 (**C**)**,** and MDA-MB-435 (**D**) cells. The black bars indicate treatment with free IPA-3 and the white bars indicate treatment with SSL-IPA-3. DMSO and empty SSL were used as vehicle controls for free IPA-3 and encapsulated IPA-3, respectively. Data are representative of three different experiments using three different passages (*n* = 3). Data are presented as the mean ± SEM; * indicates a significant (*p* < 0.05) difference between mean values of different treatments and their controls.

We previously showed that TNBC cells had a higher sensitivity to the activity of free unencapsulated IPA-3 compared to non-metastatic cancer cells (MCF-7) [38]. Treatment of metastatic TNBC cells with SSL-IPA-3 decreased MTT staining in a dose-dependent manner (Figure 2). SSL-IPA-3 did not significantly decrease MTT staining in MCF-7 cells, which was expected as these cells appear to be resistant to the activity of free IPA-3 [38].

**Figure 3:**
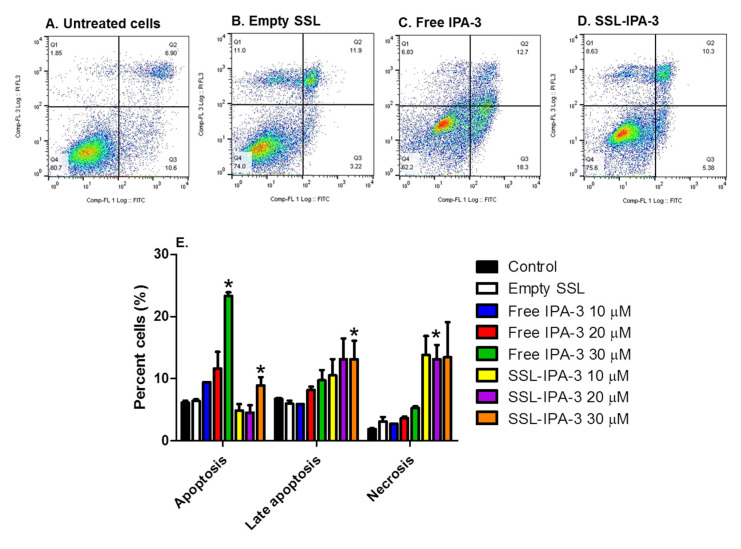
Effect of IPA-3 and SSL-IPA-3 on annexin V and PI staining in MDA-MB-468 breast cancer cells. (**A**–**D**) Scatter plots representing annexin V (x-axis) and PI staining (y-axis) in control untreated cells (**A**), cells treated with empty liposomes (**B**), and those treated with free IPA-3 (**C**) and SSL-IPA-3 (**D**) for 48 h. (**E**) Quantification of staining of annexin V and PI. Data are presented as the mean ± SEM; * indicates a significant (*p* < 0.05) difference between mean values of different treatments and their controls.

Annexin V and PI staining were measured to determine if decreases in MTT staining were a result of cell death. We focused these studies on MDA-MB-468 cells, which showed the highest sensitivity to both free IPA-3 and SSL-IPA-3. Treatment of these cells with empty liposomes did not cause significant changes in the number of cells staining positive for annexin V and/or PI when compared to control cells. Treatment of cells with free IPA-3 increased the number of cells staining positive for annexin V (Figure 3). Increases were also seen in cells staining positive for both annexin V and PI; however, free IPA-3 did not result in an increase in cells positive for PI, even at the highest concentrations tested. Similarly, SSL-IPA-3 also increased the percentage of cells staining positive for annexin V alone, as well as those staining positive for both annexin V and PI. Interestingly, and unlike free IPA-3, SSL-IPA-3 treatment increased the number of cells staining positive for PI alone, suggesting that these liposomes may also induce cell death via necrosis.

### 2.3. Effect of Secretory Responsive Liposomes Containing IPA-3 (SPRL-IPA-3) on Breast Cancer Cell Viability

**Figure 4:**
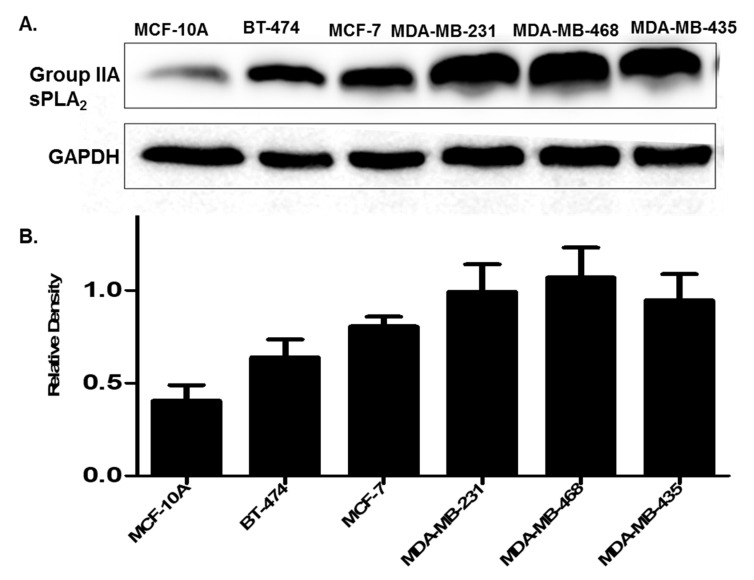
(**A**) Expression of Group IIA sPLA_2_ in breast cancer cell lines. Cells are representative of different stages of breast cancer. MCF-10A (non-cancerous), BT-474 (cancerous but non metastatic), MCF-7 (invasive non-metastatic), and MDA-MB-231, MDA-MB-468, and MDA-MB-435 metastatic triple-negative breast cancer (TNBC). (**B**) Densitometry analysis of Group IIA sPLA_2_ expression. Data are representative of three experiments (*n* = 3).

Only a few studies exist determining the expression of Group IIA sPLA_2_ in breast cancer [37,39,40]. Those studies that do exist measuring Group IIA sPLA_2_ protein expression in clinical tissues report similar levels in both primary and metastatic tumors [37]. We validated that this type of expression was also seen in the cell line panel tested (Figure 4). Further, similar to prostate cancer cells and patients, expression of Group IIA sPLA_2_ was higher in cell lines derived from highly metastatic and aggressive TNBC cells, as compared to non-cancerous (MCF-10A) and non-metastatic cells (BT-474, MCF-7). We also assessed the location of Group IIA sPLA_2_ in select cells by treating them with pronase, which can cleave and release membrane proteins. As predicted for a membrane-localized protein, pronase treatment decreased Group IIA sPLA_2_ levels in all cell lines tested (Supplementary Figure S2). Bright-field microscopy was used to verify the viability of these cells after pronase treatment to ensure that any decreases were not due to cell death from membrane rupture.

**Figure 5:**
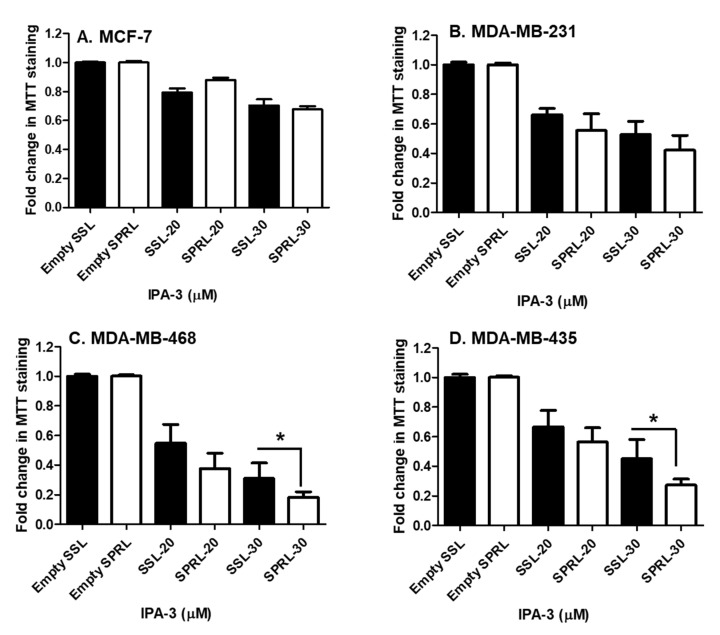
Effect of SSL-IPA-3 and SPRL-IPA-3 on MTT staining in human breast cancer MCF-7 (**A**), MDA-MB-231 (**B**), MDA-MB-468 (**C**), and MDA-MB-435 cells (**D**). The black bars indicate cells treated with SSL-IPA-3 and the white bars indicate cells treated with SPRL-IPA-3. Empty SSL and empty SPRL were used as vehicle controls. Data are representative of three different experiments using three different passages (*n* = 3). Data are represented as the mean ± SEM. * Indicates a significant (*p* < 0.05) difference between mean values of different treatments.

Having verified the expression and membrane location of Group IIA sPLA_2,_ we next compared the efficacy of SPRL-IPA-3 to SSL-IPA-3 using MTT staining. Similar to the effect of free IPA-3 and SSL-IPA-3, SPRL-IPA-3 did not decrease MTT staining in the less metastatic MCF-7 cells (Figure 5). In contrast, SPRL-IPA-3 appeared to induce greater decreases in MTT staining at the highest dose used in MDA-MB-468 and MDA-MB-435 cells.

**Figure 6:**
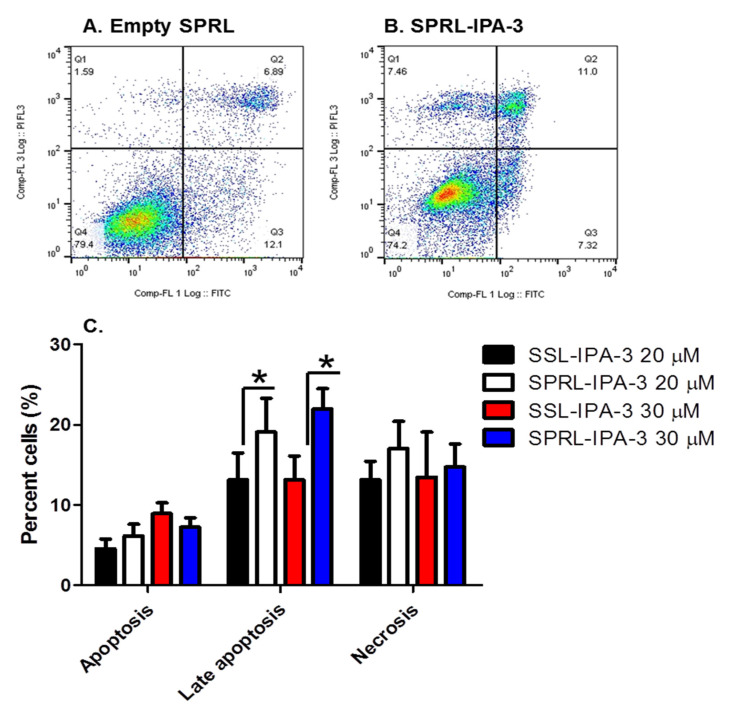
Effect of SSL-IPA-3 and SPRL-IPA-3 on annexin V and PI staining in breast cancer MDA-MB-468 cells. (**A**,**B**) Scatter plots representing annexin V (x-axis) and PI staining (y-axis) in control (**A**) and SPRL-IPA-3-treated (**B**) cells for 48 h. (**C**) Quantification of annexin V and PI staining. Data are presented as the mean ± SEM; * indicates a significant (*p* < 0.05) difference between mean values of different treatments and their controls.

We further compared the ability of SPRL-IPA-3 to induce cytotoxicity using annexin V and PI staining (Figure 6). We focused these studies on MDA-MB-468 cells as these appeared to be the most sensitive cell line tested based on decreases in MTT staining (Figure 5). Similar to free IPA-3 and SSL-IPA-3, SPRL-IPA-3 treatment increased the percentage of cells staining positive for both annexin V and PI (Figure 6). Further, SPRL-IPA-3 treatment appeared to have a significantly higher effect in inducing apoptosis as evidenced by the increased number of cells staining positive for annexin V and PI, as compared to SSL-IPA-3. An increase in the number of annexin V- or PI-positive cells was similar between SPRL-IPA-3 and SSL-IPA-3 (Figure 6C). These data suggest that SPRL-IPA-3 can induce cell death in breast cancer cell lines and the mechanism of cell death is similar to SSL-IPA-3. These data also suggest that SPRLs induce cell death at a superior efficacy in MDA-MB-468 cells.

Our data suggest that the efficacy of SPRL-IPA-3 in cells with high Group IIA sPLA_2_ is higher than that seen in cells with lower expression. To investigate whether this observation is drug-dependent, we tested the effect of SPRL loaded with doxorubicin (SPRL-Dox) in BT-474 cells, which had relatively lower levels of Group IIA sPLA_2_ expression, and MDA-MB-468 cells, which had relatively higher levels of Group IIA sPLA_2_ (Figure 4). Treatment of BT-474 cells with SPRL-Dox slightly decreased MTT staining as compared to controls. In contrast, treatment of MDA-MB-468 cells resulted in significant decreases in MTT staining, even at the lowest concentration tested (Supplementary Figure S3A). Such a finding suggests that the efficacy of both SSL and SPRL liposomes is dependent on both the encapsulated drug and the cell type.

It should be noted that SPRLs are designed to interact with sPLA_2_ and our previous studies showed that the sPLA_2_ inhibitor LY311727 did not alter the activity of SPRL-Dox [35]. This suggested that the efficacy of SPRL is independent of sPLA_2_ enzymatic activity. This result had only been shown in prostate cancer cells. Thus, we tested the ability of the sPLA_2_ inhibitor varespladib to alter the ability of SPRL-Dox to decrease MTT staining in breast cancer cells. Varespladib is more potent and selective for Group IIA sPLA_2_ than LY311727. Similar to our previous studies using the LY311727 inhibitor, varespladib pretreatment did not alter the activity of SPRL-Dox in any cell line tested, which included those with the highest expression of Group IIA sPLA_2_ (Supplementary Figure S3B–D).

## 3. Discussion

PAK-1 is an attractive therapeutic target in various cancers including breast cancer [7]. This protein is overexpressed in the early stages of breast cancer during the conversion of the normal epithelium to ductal carcinoma in situ (DCIS) [41]. Further, overexpression of PAK-1 induced malignant transformation of mammary cells in transgenic mouse models, in addition to other breast lesions such as ductal hyperplasia and formation of solid nodules [42].

There is a strong correlation between increased nuclear localization of PAK-1 and resistance to the anti-estrogen tamoxifen in breast cancer [43]. PAK-1 phosphorylates the estrogen receptor alpha (ERα), correlating to increased ER receptor expression and resistance to tamoxifen [43,44,45]. This suggests that PAK-1 inhibition may be an effective strategy to overcome tamoxifen resistance in breast cancer [43,46].

Alteration of PAK-1 expression has also been associated with hormone receptor-positive breast cancer cells and increased PAK-1 expression was associated with lymph node metastasis [47,48]. Studies using RNA interference targeting PAK-1 in breast cancer cells revealed major roles of PAK-1 in cell survival and transformation [49]. Consistent with these findings, studies using transgenic mice models showed a role for overexpressed PAK-1 in the paraneoplastic and breast carcinoma transformations [42]. In addition, PAK-1 has been suggested to be involved in the regulation of apoptosis, and it is suggested that activation and overexpression of PAK-1 protect against chemotherapeutically induced cell death [19,50,51,52,53].

Even though studies have suggested that IPA-3 may be a viable therapeutic option for cancers with altered PAK-1 expression, IPA-3 has limitations due to its poor stability and efficacy in vivo [24]. We previously addressed this limitation in prostate cancer cells using SSL-IPA-3 [32] but did not test this formulation in other cancer cell models. Our data demonstrate that both SSL-IPA-3 and SPRL-IPA-3 induce cell death in metastatic TNBC cells, which extends the possible utility of these liposomes. Previous studies from our laboratories have already demonstrated that IPA-3 exposure increased the expression and activity of apoptotic proteins such as caspase-3 and -9 [24]. We have also demonstrated the ability of both free IPA-3 and liposome-encapsulated IPA-3 to induce apoptosis as shown by TUNEL staining [32]. It is worthy to note that MDA-MB-435 has been used for many years as a metastatic breast cancer model; however, some recent genetic profiling of this cell line identified it as a melanoma cell instead [54].

The expression of sPLA_2_ and, in particular, Group IIA sPLA_2_ has been suggested as an independent prognostic factor for disease recurrence and death in human breast cancer [55]. The only study that could be found investigating the expression of Group IIA sPLA_2_ in breast cancer patients did show expression in both primary and metastatic tumors, but no correlation between clinical stages. Data in this study not only demonstrated that Group IIA was expressed in all breast cancer cells tested but also revealed increased expression in metastatic TNBC cells.

We previously reported that SPRLs containing doxorubicin (SPRL-Dox) were more effective at inhibiting prostate tumor growth as compared to SSLs containing doxorubicin [35,36]. This finding suggested that SPRLs, which were engineered to take advantage of higher expression of Group IIA sPLA_2_ in prostate cancer cells, are more efficacious than their SSL counterpart. However, SPRLs have never been validated in non-prostate cancer cell lines. As such, we tested the ability of SPRL-IPA-3 to alter breast cancer cell viability and compared their efficacy to SSL-IPA-3.

SPRL-IPA-3 demonstrated increased efficacy in cells derived from TNBC as compared to SSL-IPA-3. The only difference between these two formulations was an increase in DSPE in SPRL-IPA-3. This suggests that the increased anionic lipids allow for increased association with Group IIA sPLA_2_. It is unlikely that the increased efficacy of SPRL-IPA-3 was mediated by Group IIA sPLA_2_ activity, as varespladib, a selective Group IIA sPLA_2_ inhibitor, did not alter the efficacy of SPRL encapsulated doxorubicin. This is similar to data derived in prostate cancer cells in which LY311727, a non-selective sPLA_2_ inhibitor, also had no effect [35].

Our previous data showed that SSL and SPRL containing doxorubicin demonstrated equal potency in vitro against a panel of prostate cancer cells that had a differential expression of Group IIA sPLA_2_; however, SPRL were 2-fold more effective at inhibiting growth in xenograft mouse models of prostate cancer [35]. One reason for the differences between these two studies was that previous formulations contained doxorubicin. This hypothesis is supported by data in Supplementary Figure S3 demonstrating increased efficacy of SPRL-Dox in MDA-MB-468 (high sPLA_2_ expression) cells as compared to BT-474 (low sPLA_2_ expression). A direct comparison of SPRL-IPA-3 and SPRL-Dox is not practical given the different molecular targets of these drugs; however, these data suggest that both formulations may be viable therapeutics for the treatment of triple-negative breast cancer. Further, these studies are the first to test the efficacy of either formulation in breast cancer cells.

## 4. Materials and Methods

### 4.1. Chemicals, Reagents, Cell Lines, and Cell Culture

The human breast cancer cell lines MCF-10A, BT-474, MCF-7, MDA-MB-231, MDA-MB-468, and MDA-MB-435 were purchased from ATCC (Manassas, VA, USA). RPMI medium supplemented with 10% (*v*/*v*) Fetal Bovine Serum (FBS) and 1% (*v*/*v*) penicillin/streptomycin antibiotics was used to culture cells in 37 °C and 5% CO_2_ incubators.

Phospholipids used in liposomes (Table 1) were purchased from Avanti Polar Lipids, Inc. (Alabaster, AL, USA). IPA-3 was obtained from Tocris Bioscience (Bristol, UK). Cholesterol, MTT [3-(4,5-dimethylthiazol-2yl)-2,5-diphenyltetrazolium bromide], and annexin V/PI kit were purchased from ThermoFisher Scientific (Waltham, MA, USA). All other chemicals were obtained from Fisher Scientific (Pittsburgh, PA, USA).

### 4.2. Preparation of Stealth or Sterically Stabilized IPA-3 (SSL-IPA-3) and Secretory Phospholipase Responsive IPA-3 Liposomes (SPRL-IPA-3)

Liposomes were prepared as described previously [32,35,36]. Liposome compositions are shown in Table 1. Briefly, Cholesterol (5 µmol/mL), phospholipids including DSPC (8 or 9 µmol/mL), DSPE–PEG (1 µmol/mL) in chloroform, DSPE (1 µmol/mL) in chloroform, and IPA-3 (4 µmol/mL in ethanol) were mixed, and organic solvents were evaporated under vacuum using a rotary evaporator (Buchi Labortechnik AG, Postlfach, Switzerland). The formed thin films were hydrated and suspended in phosphate saline buffer (PBS) followed by five cycles of freeze–thaw cycles and then high-pressure extrusion through a Lipex extruder repeated at least five times (Northern Lipids, Inc., Burnaby, BC, Canada) using double-stacked polycarbonate membranes (80 nm, GE Osmonics, Trevose, PA, USA). Dialysis in 10% (*w*/*v*) sucrose overnight was conducted to eliminate unencapsulated IPA-3 and lipids. A dynamic light scattering particle size analyzer was used to determine the size of liposomes (Zetasizer Nano ZS, Malvern Instruments, Enigma Business Park, Grovewood Road, Malvern, Worcestershire, UK). The size of the liposomes was further confirmed using tandem electron microscopy (TEM) imaging (Georgia Electron Microscopy, Athens, GA USA).

### 4.3. In Vitro Activity of Liposomal IPA-3 on MTT Staining

The efficacy of free and liposomal IPA-3 was determined in four cell lines representing different stages of breast cancer (MCF-7, MDA-MB-231, MDA-MB-468, and MDA-MB-435) using the cellular metabolic activity MTT assay [56]. Cells were seeded and then treated with free IPA-3, SSL-IPA-3 for 24 h. DMSO (vehicle for IPA-3) and empty liposomes (vehicles for encapsulated IPA-3) were used as controls to treat the different cell lines. The treated cells were incubated for 24, 48, and 72 h. MTT was added at each time point at a final concentration of 0.25 mg/mL and plates were kept in a 5% CO_2_ incubator at 37 °C for 2 h. Plates were then shaken for 15 min and absorbance of each well including control and blank wells was measured at 590 nm using a Spectra Max M2 plate reader (BMG Lab Technologies, Inc., Durham, NC, USA).

### 4.4. In Vitro Cytotoxicity of Liposomal IPA-3 as Assessed by Annexin V/PI Staining

Annexin V and propidium iodide (PI) staining was assessed using flow cytometry to confirm MTT staining and to assess the mechanism of cell death. The method used was as previously described [32,57]. Briefly, cells were seeded and allowed to grow for 24 h prior to treatment with free IPA-3, SSL-IPA-3, SPRL-IPA-3, or empty liposomes. After 48 h, cells were collected, washed with PBS, and then stained with Alexa Fluor^®^ annexin V-FITC and PI (100 µg/mL) for 15 min according to the manufacturer’s protocol. Annexin V and PI staining were quantified using a Dako Cyan ADP 9 color flow cytometer (Beckman Coulter, Inc., Miami, FL, USA). For each measurement 20,000 events (cells) were counted.

### 4.5. Immunoblot Analysis

Cell lysates from different cell lines were collected in RIPA buffer, which contained a protease inhibitor cocktail (Santa Cruz Biotechnology, Inc., Santa Cruz, CA, USA). The concentration of proteins in different samples was determined using the BCA assay. Cell lysates were first separated using gel electrophoresis and then transferred to nitrocellulose membranes and blocked for 2 h. The nitrocellulose membranes were incubated with a rabbit sPLA_2_ IIA antibody (Cell Signaling Technology, Danvers, MA, USA) at a dilution of 1:500 in TBS-T with 1% (*w*/*v*) BSA overnight. The antibody against GAPDH (Santa Cruz Biotechnology Inc., Santa Cruz, CA, USA) was used at a dilution of 1:4000 in 1% (*w*/*v*) BSA in TBS-T for 1 h. Membranes were then incubated with the appropriate peroxidase-conjugated secondary antibody (Promega, Madison, WI, USA) used at a dilution of 1:2500. Membranes were then washed with TBS-T three times for 10 min. Bands were developed using chemiluminescent substrates for horseradish peroxidase (Thermo Scientific, Waltham, MA, USA) and visualized using a Fluorchem SP digital imager (Alpha Innotech, San Leandro, CA, USA). Densitometry to quantify immunoblot bands was performed using the National Institutes of Health Image J software (ImageJ, U. S. National Institutes of Health, Bethesda, Maryland, USA).

### 4.6. Proteolytic Digestion of Cell Surface Proteins

Metastatic TNBC cells (MDA-MB-231, MDA-MB-468, and MDA-MB-435) were treated with pronase, which is reported to induce the cleavage of membrane proteins [58]. Briefly, subconfluent cell monolayers were incubated with 0.1% pronase in serum-free media for 20 min at 37 °C. Cells were then collected as single-cell suspensions and washed in PBS by centrifugation at 800× *g* for 5 min at 4 °C. Cell viability following pronase treatment was checked under a bright field microscope. Cells were then prepared for immunoblot analysis. Cells not treated with pronase were used as control.

### 4.7. Statistical Analysis

All experiments were repeated at least three times (*n* = 3) and cells were isolated from three different passages. The average of all replicates ± SEM are shown. Data with confirmed Gaussian distribution were compared using unpaired Student’s *t*-test. A nonparametric test such as the Mann–Whitney test was used if data did not have Gaussian distribution using GraphPad Prism software (La Jolla, CA, USA). The significance level (alpha) was set at 0.05 (marked with symbols (*)).

## 5. Conclusions

These data demonstrate the novel finding that a liposome formulation designed to target PAK-1 and Group IIA sPLA_2_ demonstrated increased efficacy against TNBC cells as compared to cells derived from non-metastatic breast cancer tissues. These data also suggest that the efficacy of the Group IIA sPLA_2_ responsive liposomes (SPRL-IPA-3) is independent of the enzyme activity and that the efficacy of these liposomes is dependent on multiple factors, including the encapsulated drug and the expression of PAK-1 (the target of IPA-3) and/or Group IIA sPLA_2_ (the target of SPRL). These data provide additional information regarding the molecular determinants of liposome efficacy and suggest that developing liposomes that have dual-target proteins known to be expressed in metastatic cancer can increase efficacy and identify novel therapeutics.

## Figures and Tables

**Table 1:** Sterically Stabilized Liposomes (SSL) and Secretory Phospholipase Responsive Liposomes (SPRL) composition. Phospholipids used are: 1,2-distearoyl-*sn*-glycero-3-phosphatidylcholine (DSPC), 1,2-distearoyl-*sn*-glycero-3-phosphatidylethanolamine (DSPE), 1,2-distearoyl-*sn*-glycero-3-phosphoethanolamine–N-poly(ethyleneglycol) 2000 (DSPE-PEG).

	Lipid Composition (Molar Ratio) in Liposomes
SSL	DSPC:Cholesterol: DSPE-PEG = 9:5:1
SPRL	DSPC:Cholesterol: DSPE:DSPE-PEG = 8:5:1:1

## Supplementary Materials

Supplementary Materials can be found at https://www.mdpi.com/1422-0067/21/24/9396/s1.

## Abbreviations

BCS	breast-conserving surgery
Cdc42	cell division control 42 protein
DLS	dynamic light scattering
DSPC	1,2-distearoyl-*sn*-glycero-3-phosphatidylcholine
DSPE	1,2-distearoyl-*sn*-glycero-3-phosphatidylethanolamine
DSPE-PEG	1,2-distearoyl-*sn*-glycero-3-phosphoethanolamine–N-poly(ethyleneglycol) 2000
IC50	half maximal inhibitory concentration
IPA-3	inhibitor targeting PAK-1 activation-3
MTT	3-(4,5-dimethylthiazol-2yl)-2,5-diphenyltetrazolium bromide
PAK-1	p21 activated kinase-1
PDI	polydispersity index
SEM	standard error of the mean
SSL	sterically stabilized liposomes
SL	stealth liposomes
SPRL	secretory phospholipase A2 responsive liposomes
sPLA_2_	secretory phospholipase A2
Rac	Ras-related C3 botulinum toxin substrate 1
TEM	tandem electron microscopy
TNBC	triple-negative breast cancer
