# Supplementary material for: Liposomes Targeting P21 Activated Kinase-1 (PAK-1) and Selective for Secretory Phospholipase A2 (sPLA2) Decrease Cell Viability and Induce Apoptosis in Metastatic Triple-Negative Breast Cancer Cells
Source: Int J Mol Sci. 2020 Dec 10;21(24):9396. doi: 10.3390/ijms21249396 (PMC7764208; doi:10.3390/ijms21249396)
Supplement: Supplementary file 1 [file ijms-21-09396-s001.pdf]

Supplementary Materials

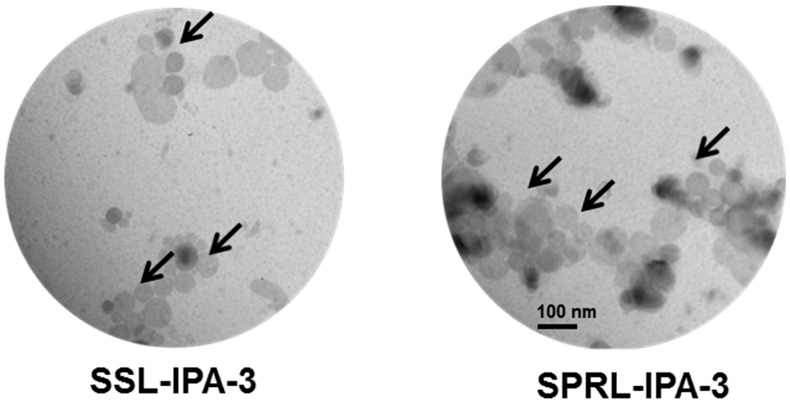

Supplemental Figure S1. Tandem Electron Microscopy (TEM) of stealth or sterically stabilized IPA-3 Liposomes (SSL-IPA-3 and SPRL-IPA-3). Samples of liposomal suspensions were placed on a carbon-coated copper grid and dried overnight and then stained using uranyl acetate. Samples were imaged the next day using TEM.

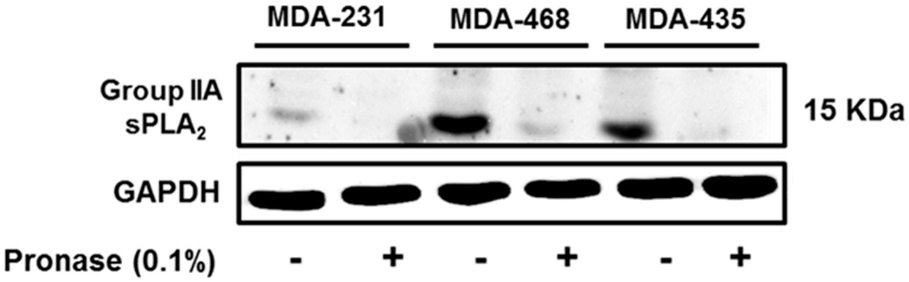

Supplemental Figure S2. Effect of pronase treatment on the expression of Group IIA $sPLA_2$ in metastatic TNBC MDA-231, MDA-468, and MDA-435 cells. .

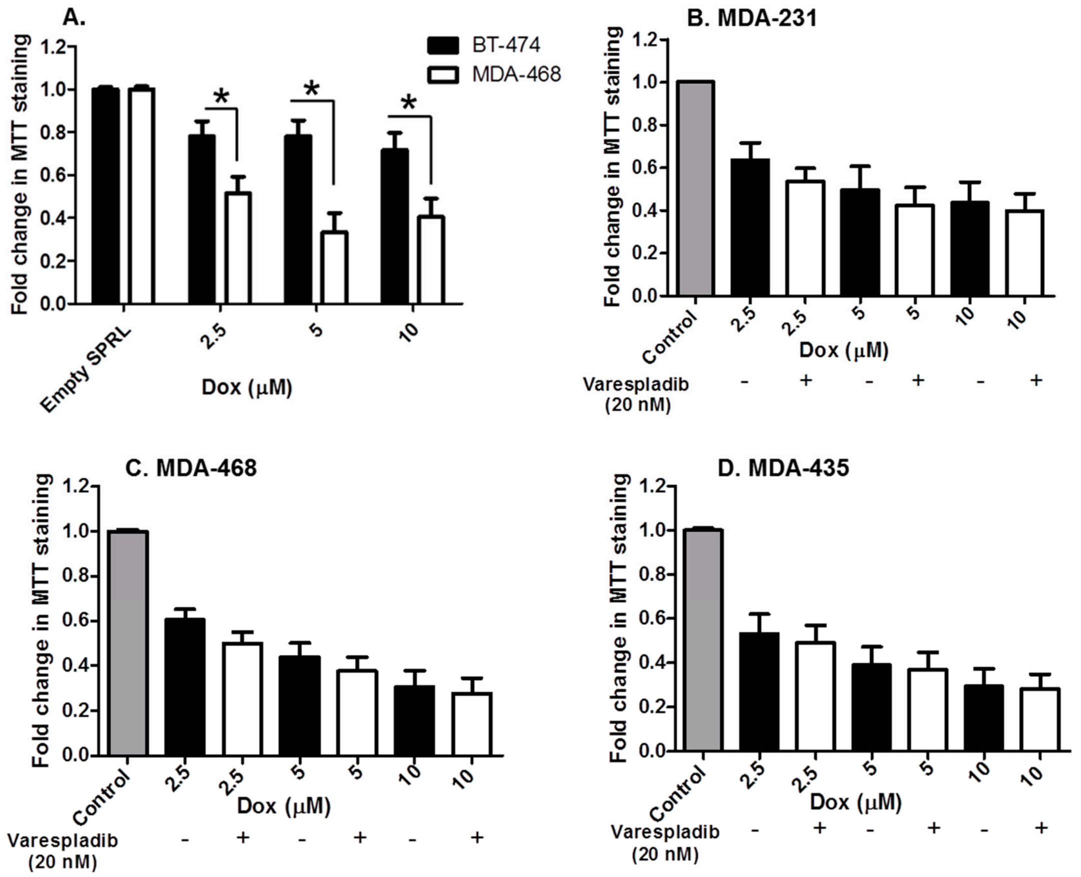

Supplemental Figure S3. (A) Effect of SPRL-Dox on MTT staining in the low Group IIA $sPLA_2$ expressing human breast cancer BT-474 cells, and the high Group IIA $sPLA_2$ expressing MDA-468 cells 48 hrs after treatments (n = 3). The black bars indicate the effects of SPRL-Dox in BT-474 cells and the white bars indicate the effects of SPRL-Dox in MDA-468 cells. Empty SPRL were used as vehicle controls for encapsulated Dox. **(B-D)** Effect of a Group IIA $sPLA_2$ inhibitor (varespladib) on the efficacy of SPRL-Dox. MTT staining was assessed in human metastatic breast cancer MDA-231 **(B)**, MDA-468 **(C),** and MDA-435 cells **(D)** 48 hrs after treatments (n = 3). The black bars indicate the effects of SPRL-Dox without pretreatment with varespladib. The white bars indicate the effect of SPRL-IPA-3 in cells pretreated with varespladib. Empty SPRL were used as controls and are shown in grey bars. Data are representative of three different experiments using three different passages (n = 3). Data are presented as the mean ± SEM; *Indicates a significant ($p < 0.05$) difference between mean values of different treatments.
